# The Prolongation of Life

**Published:** 1906-05-12

**Authors:** 


### The Hospital

A Journal of

The Medical Sciences and Hospital Administration.

Vol. XL.—No. 1,024. SATURDAY, MAY 12, 1906.

### The Prolongation of Life.

In his small volume "On Means for the Prolongation of Life," Sir Hermann Weber has written shrewdly and with due scientific reserve on a subject which only too often has been the opportunity for sensational and dramatic announcements. His essay is manifestly the outcome of much careful and thoughtful observation and reflection, and to describe it as common sense illuminated by the knowledge and experience of a wise physician is both to pay it a high compliment and also to mark its range and value. No one knowing its author's record will expect to find in it either short cuts to health or a narrow dogmatism which solves all the difficult problems of life and health by the continual repetition of a simple and confident formula. On the contrary, one of the most pleasing features of the book is the recurring attempt to find in physiological truths the practical guidance necessary for the attainment and conduct of a healthy and vigorous life. The ambition is not merely length of days, but a useful existence, and in endeavouring to promote both these ends Sir Hermann Weber seems to us to have made a substantial contribution to practical therapeutics, including in that term all the agencies and influences by which health of body and mind may be promoted. After all, the essential principle which guides studies of this order must be a presentation of the necessity on the part of the body to adjust itself to its environment, or, perhaps, still better, to secure a harmony between its internal and external relations. The difficulty here, as in many other cases, is to translate a sound principle into wise action—a difficulty which is all the greater when wisdom consists in a multitude of sustained and small observances rather than in some large and decisive course of conduct. To those who see in uric acid on the one hand, or "hyperpyræmia" on the other, the unvarying and constant explanation of most of the physiological disasters which make shipwreck of human health, counsel and direction come with a ready flow. But Sir Hermann Weber has not so read the complex problem of life. Hence his guidance has a wider range. It includes, indeed, directions on all points which bear on the culture of health, and this more especially at the time when the natural vigour of manhood has begun to pass into its inevitable decline. It certainly is an impressive fact that, while, during the last 30 or 40 years, the death-rate of children and of adults under 45 years of age has been sensibly reduced, no such diminution can be detected in the death-rate of adults of 50 years of age and upwards. The problem here, then, is to increase the longevity of individuals. In Sir Hermann Weber's view there is reason to anticipate that a study of the operation of the various agencies which affect the prolongation of life will probably lead, in the course of time, to an elevation of the limit of human existence to one hundred years, or even to a longer period. What these agencies are, how they operate, and how those of beneficial influence are to be encouraged, and those with an opposite tendency checked, is the subject-matter of his book; and all those who take a comprehensive view of their duties as medical advisers will be well advised to read it. They will not find in its pages anything which can be described as startlingly original, though there are many very helpful and practical suggestions on such subjects as diet, exercise, sleep, and alcohol. Sir Hermann Weber believes strongly that if a man will enjoy a happy and useful old age he must devote time and attention to the care of his health in his younger days, and without endorsing every detail we believe his counsel towards this end to be eminently sane and wise. The cultivation of a "fresh-air" life, the regulation of the quantity and nature of the diet, the adjustment of exercise to bodily capacity, the use of baths—these and such like questions obviously have the most intimate bearing on health, and on them the physician should be prepared to afford his patients counsel and help. Possibly the virtues of drugs just now are somewhat unduly depreciated, but whether this is so or not, no one will question the importance of the agencies which have just been enumerated. Their study cannot too forcibly be impressed on the profession, and all efforts in this direction are to be cordially welcomed. To add ten years to the life of an individual is no small triumph, and the effect on human history of a general extension of the duration of man's years can hardly be exaggerated. An ambition directed to this end is hardly likely to meet with any sensational recognition or reward, but none the less it may be a substantial contribution to the welfare of mankind.

